# Pulmonary Eosinophils at the Center of the Allergic Space-Time Continuum

**DOI:** 10.3389/fimmu.2021.772004

**Published:** 2021-11-15

**Authors:** Sjoerd T. T. Schetters, Martijn J. Schuijs

**Affiliations:** ^1^ Department of Internal Medicine and Pediatrics, Ghent University, Ghent, Belgium; ^2^ Laboratory of Immunoregulation and Mucosal Immunology, VIB-UGent Center for Inflammation Research, Ghent, Belgium; ^3^ Cancer Research Institute Ghent, Ghent, Belgium

**Keywords:** eosinophils, immunology, asthma, lung, allergic airway inflammation, innate immunity

## Abstract

Eosinophils are typically a minority population of circulating granulocytes being released from the bone-marrow as terminally differentiated cells. Besides their function in the defense against parasites and in promoting allergic airway inflammation, regulatory functions have now been attributed to eosinophils in various organs. Although eosinophils are involved in the inflammatory response to allergens, it remains unclear whether they are drivers of the asthma pathology or merely recruited effector cells. Recent findings highlight the homeostatic and pro-resolving capacity of eosinophils and raise the question at what point in time their function is regulated. Similarly, eosinophils from different physical locations display phenotypic and functional diversity. However, it remains unclear whether eosinophil plasticity remains as they develop and travel from the bone marrow to the tissue, in homeostasis or during inflammation. In the tissue, eosinophils of different ages and origin along the inflammatory trajectory may exhibit functional diversity as circumstances change. Herein, we outline the inflammatory time line of allergic airway inflammation from acute, late, adaptive to chronic processes. We summarize the function of the eosinophils in regards to their resident localization and time of recruitment to the lung, in all stages of the inflammatory response. In all, we argue that immunological differences in eosinophils are a function of time and space as the allergic inflammatory response is initiated and resolved.

## Introduction

Eosinophils represent a minority population of peripheral leukocytes of the innate immune system. They are largely evolutionary conserved and classically considered terminally differentiated end-stage cells (1). Eosinophils develop in the bone marrow from myeloid precursors under the influence of interleukin (IL)-5. Although IL-5 is critical for eosinophil differentiation, priming, and survival, other cytokines, as IL-3 and granulocyte-macrophage colony stimulating factor (GM-CSF) also promote eosinophil differentiation (2). Upon release into the circulation eosinophils are present in the peripheral blood for a few hours; however, they can survive in tissues for several weeks and adopt tissue-specific homeostatic phenotypes (3). The ability of eosinophils to remain in tissues for extended periods of time suggest they have a necessary role in homeostasis or preventing disease (2). As postulated in the Local Immunity And/or Remodeling/repair (LIAR) hypothesis, by James Lee, eosinophils can be considered intrinsically homeostatic cells that are associated with sites characterized by high cell proliferation/turn-over and cell death (4). Indeed, eosinophils are, under homeostatic conditions, distributed in many organs like the lung, spleen, and gastrointestinal tract, as well as in the blood, lamina propria and adipose tissue (5). As such, these cells are proposed to have a physiological function in each of these different organs, which is strengthened by evidence on the existence of multiple tissue specific subtypes of eosinophils based on distinct surface marker expression and functional characteristics (6, 7). Although they are equipped with an arsenal of pre-formed inflammatory mediators and have the ability to produce several cytokines, eosinophils are most well-recognized for their pivotal role in the inflammatory pathology of a broad range of diseases, including parasitic infections and allergic disease, such as food allergy, asthma, and atopic dermatitis (8). Whether eosinophils are also involved in the resolution phase of these inflammatory afflictions is largely unknown. In general, the immune response after acute inflammation and the accompanying tissue damage is meant to resolve inflammation, repair tissue and re-establish tissue homeostasis.Therefore, it is essential to accurately study the function of immune cells, not only regarding their location, but also include their temporal exposure to different microenvironments at that location. Here we emphasize the need to define eosinophils during acute, late, and chronic inflammatory responses, as well as resolution in lung inflammation in regards to both time and space.

## Eosinophils in Maintenance of Immunological Homeostasis

At birth very few eosinophils are present in the lungs of mice, however they are recruited by IL-5 from type-2 innate lymphoid cells (ILC2) under the influence of epithelium-derived IL-33 coinciding with the alveolarization phase at post-natal day (PND) 3. After which they rapidly increase in number, peaking on PND14, before the eosinophils decline again after weaning (9). Importantly, eosinophil adopt a type 2 activated immune phenotype during this phase (10). In humans, eosinophils have been shown to be present as early as fetal thymic development (11). From birth onwards the lungs are constantly exposed to a variety of airborne particles and these insults typically result in clearance without acute inflammation, as well as antigenic tolerance. Several studies, in both mice and humans (12, 13), have shown that eosinophils spend between 3 and 24 hours in circulation, however their half-life in the lung is prolonged to about 36 hours (3). Additionally, homeostatic lung eosinophils express several genes, like *Runx3, Serpinb1a*, and *Ldlr*, that are implicated in the maintenance of lung immune homeostasis and negative regulation of T helper cell type 2 (Th2 cell) responses (14). In line with these observations, studies in eosinophil-deficient mice have revealed that sensitivity to house dust mite (HDM) is increased in the absence of eosinophils (14). The unique capability of lung homeostatic eosinophils to prevent Th2-driven allergic airway inflammation has been linked to their ability to inhibit the maturation of allergen-loaded dendritic cells (DCs) (14). However, seeing that eosinophils are central to the alveolarization phase early in life, the widespread use of congenital ΔdblGATA mice and PHIL mice, that both lack eosinophils, may significantly confound experiments performed in adult life. It is still unclear how the absence of eosinophils at birth will impact later respiratory challenges like allergens, bacterial and viral infections.

In the steady state adult lung, Mesnil et al. have identified a small population of tissue-resident eosinophils (rEos). These eosinophils are found to express distinct surface markers like the L-selectin receptor CD62L, that is distinct from “inflammatory” eosinophils (iEos) appearing after allergic inflammation. Even though rEos express the IL-5 receptor, their presence in the lung seem to be IL-5 independent and may promote the development of Th1 immunity by impairing the ability of DCs to induce Th2 immunity (14). In contrast, earlier findings of Nussbaum et al. suggest that basal eosinophilopoiesis and accumulation of eosinophils in tissues is dependent on ILC2-derived IL-5 (15). These apparent contradictions on the role of IL-5 in basal conditions of tissue-eosinophilia highlight the need for a better characterization of the precise role these lung-resident eosinophils have, especially when translating these findings to the human lung (16). Recently, an intra vital microscopy study in mice showed patrolling eosinophils in the lung vasculature, which were differentially activated after stimulation with ovalbumin (OVA)-allergen, suggesting these resident cells to be reactive to allergenic insults (17). Activation of eosinophils to airborne allergens is often studied with purified molecules, like: IL-33, papain, and Aspergillus protease. The use of these type-2 inducing agents allow for a reductionistic experimental system to investigate airway allergy. However, real-life allergens (e.g. HDM) better recapitulate the spatiotemporal interplay between innate and adaptive immunity, including the pleiotropic function of eosinophils epitomized in this review. With new tools becoming available homeostatic- or resident-lung eosinophils can be further characterized and questions on their contribution to the maintenance of homeostasis and tolerance in the lung and the presence of different eosinophil subpopulations can be addressed.

## Eosinophils Promote Th2 Differentiation During Sensitization (Pre-Challenge)

The first encounter with allergens, like; HDM, and the absence of type 1 inflammatory signals in early life (“hygiene hypothesis”) – sets the stage for allergic pathology later in life (18). It is now clear that the airway epithelial cells (ECs) play an important role in the induction of allergen-induced inflammatory responses (19). Not only can epithelial cell damage be seen in all phenotypes of asthma, changes in EC function can be observed at very young age, cumulating to the idea that ECs may play a role in the initiation of asthma in early life (20, 21). The link between epithelial barriers and eosinophils is supported by their preferred association with epithelial barrier tissues, where foreign antigens are most often encountered (6). Amongst these structural barrier cells are pulmonary neuroendocrine cells (PNECs) that are specialized tissue-resident neuroendocrine cells in the airway epithelium (22). PNECs can be innervated by both parasympathetic and sympathetic neuronal fibers (23, 24). With close proximity to steady-state immune cells, like ILC2, PNECs have the ability to amplify allergen-induced immune cell recruitment, including eosinophils (25). Interestingly, eosinophils in turn have been shown to contribute to increased nerve density and airway nerve remodeling which serves as a key mechanism for increased irritant sensitivity and exaggerated airway responsiveness (26).

For the initiation of antigen-specific Th2 responses in the lung, conventional DC2s (cDC2s) need to migrate to the draining lymph nodes, a process augmented by ILC2-derived IL-13, mast cell-derived TNF, epithelial cell-derived GM-CSF, and by type-1 interferon (27–29). Although unclear in the pulmonary setting, in the murine intestine it is proposed that eosinophils play an important role in the activation of DCs and their migration to the draining lymph nodes (30). Eosinophils have also been shown to produce an antimicrobial protein, eosinophil-derived neurotoxin (EDN), that effectively recruits and activates cytokine producing DCs, thereby enhancing Th2 immune responses (31–33). Besides promoting DC activation, murine intestinal and lymph node eosinophils have been reported to express antigen presentation machinery, including; MHC-II, costimulatory molecules CD80 and CD86, and migrate to the draining lymph nodes in a CCR7-dependant manner (34–36). Interestingly, human peripheral blood eosinophils exhibit very low to undetectable levels of MHC-II, whereas class-II expression is observed on airway eosinophils (37, 38). Eosinophils are observed within the T cell zone of the draining lymph nodes, have the ability to present antigen, and express transcripts for IL-4 and IL-13. However, the low number of these cells in the lymph nodes suggest that eosinophils have a minor role as antigen presenting cells and instead may be required for the accumulation of DCs within the lymph nodes and subsequent antigen-specific T effector cell production (39). Interestingly, these effects were independent of MHC-II expression on eosinophils, again proposing an accessory role for eosinophils in the process of T cell stimulation. Moreover, human blood-derived eosinophils have been shown to induce DC maturation by physically interacting with DCs in the presence of bacterial pathogen-associated molecular patterns (PAMPs) (40).

Together, these data demonstrate that eosinophil-derived products can promote Th2 inflammation *via* DC regulation during the sensitization phase to an allergen. At the same time, lymph node eosinophils actively suppress DC-induced Th17 and Th1 responses, thereby promoting Th2 polarization (39). It should be noted that timed depletion of eosinophils using iPHIL mice in the sensitization phase of HDM or OVA allergic airway inflammation did not affect the outcome of type 2 immunity or lung function following allergen challenge, suggesting that eosinophils may have a more subtle effect on downstream adaptive immunity (41). In all, the process of allergic sensitization, aided by eosinophils, results in the proliferation of Th2 cells, the appearance of class-switched plasma cells producing allergen-specific IgE, and the presence of IgE/FcϵR1-intraepithelial mast cells in the lung.

## Phases of Allergic Lung Inflammation

**Figure 1:**
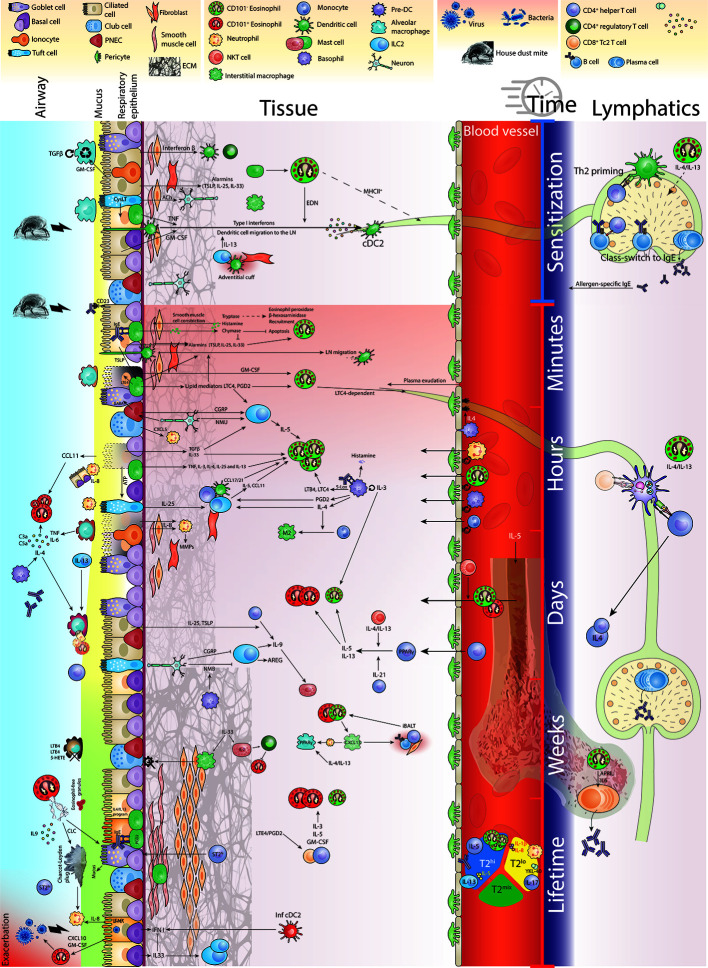
Eosinophil function in time and space during allergic airway inflammation.

Any inflammatory response is subject to critical changes through space and time, especially in damage-prone tissues like the lungs (42, 43). The allergic inflammatory response has been classified in terms of three temporal phases of inflammation, the acute, late, and chronic phase (44). Even though this paradigm is now well accepted, surprisingly little is known about the differences in eosinophil functioning during these distinct phases of allergic inflammation. Indeed, complete eosinophil-deficient animals do not distinguish between differential functioning in these phases, significantly hampering understanding of the exact role of eosinophils. For example, *in vitro* exposure of murine blood-derived eosinophils to a certain set of cytokines defines their phenotype in the lung when adoptively transferred *in vivo (* 45 *)*. Indeed, type 2 cytokines known to affect eosinophils like IL-4, IL-5 and IL-13, chemokines like CCL11 and lipid mediators, like cysteinyl leukotrienes (CysLTs) are produced by different cell types, in different locations and at different time points during the allergic inflammatory process (19). In an effort to holistically address the multi-wave inflammatory response, Walsh and colleagues constructed a network model of allergic airway inflammation that was supported by experimental perturbation experiments (46). They reported early induction of airway hyperresponsiveness (AHR) relied on mast cells in the early phase and on Th2 cells and eosinophils in the late phase. Interestingly, IL-13 seemed to differentially affect AHR in a distinctive manner through time. Other efforts are now being made to conceptualize and visualize these time-dependent inflammatory processes (47). In this review, by visualizing the allergic airway response in space and time in **Figure 1**, we aim to illuminate the heterogeneity of environments and molecular input that govern eosinophil functioning in a spatiotemporal manner.

### Acute Phase (Minutes to Hours)

In the lower airways, alveolar macrophages (AMs) are the main immune cell type encountering airborne particles, the lung-resident commensal microbiome and tasked with maintaining homeostasis (48). They actively patrol the alveoli in homeostasis and develop under the influence of epithelial-derived TGFβ and GM-CSF (49–51). As such, it is not surprising that these cell types are found to behave distinctly different in the allergen-challenged lung (52). As the inflammatory response develops and the alveolar space is intruded by other immune cells, like eosinophils, the concerted immune response is highly dynamic. At first, for the AM to initiate and support inflammation, it needs multiple molecular cues to override the TGFβ-driven tolerogenic phenotype (48, 53). Loss of AMs results in exacerbated cellular and humoral type 2 immunity (54). Recognition of HDM by AMs is mediated by Dectin-2 and results in the downstream production of CysLTs (55, 56). Alveolar macrophages readily phagocytose airborne particles and it is now recognized that the phagocytic capacity of these macrophages is dysfunctional in asthmatic patients (52). Interestingly, phagocytosis of apoptotic cells suppressed HDM-induced allergic lung inflammation (57). As the clearing capacity of macrophages is reduced, allergens like HDM can further induce epithelial responses.

Allergen-induced epithelial responses initiate the production of the cytokines IL-33, IL-25, and thymic stromal lymphopoietin (TSLP) that are released upon epithelial activation or damage. In the (naïve) lung, a wide variety of cells respond to these cytokines, including ILC2, DCs, macrophages, mast cells, basophils, and eosinophils (58). Activation of these diverse cell types leads to reciprocal interactions and the release of additional mediators. Especially ILC2 have been shown to coordinate eosinophilia in response to allergen, since they localize and migrate in close proximity to epithelial cells (59). Several cytokines can activate ILC2 in the early stage, epithelial-derived IL33 and TGFβ (60), as well as basophil-derived IL-4 (61), and tuft cell-derived IL-25 and CysLTs (62), of which the end product LTE4 is even detectable in the bronchoalveolar lavage fluid and urine of patients with asthma exacerbations (63). ILC2-derived IL-5 will act as an early mediator of eosinophil differentiation and hematopoiesis. At the same time, ILC2-derived IL-13 enhances the expression of eotaxins that assist with eosinophil recruitment (62). In turn, eosinophils can maintain ILC2 activation through the release of IL-4. Moreover, eosinophils can directly bind IL-33, inducing a wide range of transcripts supporting eosinophil activation (64). Whereas, TSLP has a major role in the recruitment of eosinophil into the respiratory tract (65), it also induces pro-survival mechanisms through direct binding of TSLP by its receptor on eosinophils (66). Besides controlling eosinophil numbers, ILC2 also license DCs to trigger adaptive Th2 cell responses (67). Furthermore, epithelial cells in the allergic lung produce GM-CSF, which controls the recruitment and survival of eosinophils in the lung directly (68). It has further been shown that activation of the NF-κB pathway in the epithelial lineage is crucial for the downstream allergic immune cascade in response to HDM (69) and interference in this cascade early in life can prevent the onset of allergic disease (18).

In recent years it has been demonstrated that large functional heterogeneity exists within the epithelial cell lineage at distinct areas of the respiratory system. Specialized tuft cells contribute to type 2 immune responses and eosinophilia through the production of IL-25 and CysLTs (70, 71). When tuft cells recognize the epithelial stress trigger ATP, they release LTC4, LTD4 and LTE4, which can augment the sensitivity of ILC2 to type 2 inflammatory mediators (72, 73). PNECs are solitary cells in the epithelium of the upper airways and function as chemosensors to respond to changes in oxygen, mechanical- and chemical stimuli by producing neuroactive mediators (22, 74). Mice lacking PNECs have been shown to exhibit reduced allergic inflammation and eosinophilia as ILC2 become less activated (75). In humans, PNECs are found to be increased in number in asthma patients, producing the neuropeptide CGRP and the neurotransmitter GABA increasing ILC2-derived IL-5 and promoting goblet cell hyperplasia, respectively (75). Activation of steady state ILC2 can be further stimulated by the neuron-derived neuromedin U, especially in the presence of IL-25 (76, 77). The specific location of these neuroimmune cell units in the lung and the temporal activation of these early response hubs in allergic inflammation will need further investigation in relation to whether and how they have direct effects on eosinophil activation (78). Importantly, allergen detection may not only be constrained to the apical side of the epithelial layer and its associated cells, as epithelial CD23 (the low-affinity IgE receptor) can bind and transcytose allergen-specific-IgE, resulting in increased allergic inflammation (79).

After the lung is sensitized to the allergen in a type-2 dominant manner, intraepithelial mast cells are primed by expressing FCϵR1 binding allergen-specific IgE. Upon allergen stimulation, membrane-bound IgE clusters FCϵR1 on mast cells leading to immediate degranulation and the release of pre-formed vesicles filled with histamine and enzymes like tryptase and chymase (80). A reciprocal relationship exists between mast cells and eosinophils, with mast cells supporting eosinophil survival and activation by secreting IL-5. Genetic knockout of mast cells reduced eotaxin levels in the lung of HDM challenged mice and impaired eosinophil recruitment (81). Eosinophil-derived MBP in turn directly activates mast cells and basophils, releasing histamine and TNF (2). It has now been established that mast cell-derived acute phase proteases modulate asthma pathology (reviewed in (82)). Moreover, histamine has multiple direct asthma-related effects in the lung, including; plasma exudation due to increased vascular permeability, release of mucus, and constriction of small respiratory passages (83). Tryptase levels in blood and airway fluid are elevated in asthma patients and correlate with disease severity (84). Interfering with tryptase using antagonistic antibodies reduced mast cell activation and the use of tryptase inhibitors or serine protease inhibitors reduced eosinophil infiltrates (85, 86). Besides, human β-tryptase has been shown to enzymatically inhibit eotaxin and RANTES function, possibly affecting eosinophil recruitment (87). Additionally, human peripheral blood eosinophils respond to enzymatically active tryptase by the release of eosinophil peroxidase and beta-hexosaminidase (88), although it remains to be determined whether the lung resident eosinophils in mice would contribute to the acute phase inflammatory response after release of tryptase. Chymase has been assigned a plethora of asthma-related activities, including increasing mucus production, modification of extracellular matrix and modulation of cytokines like IL-33, IL-4, and IL-1β (89). However, exploration of chymase (specifically, the direct mouse homologue Mcpt4) in murine models of asthma, suggests a protective role in pathology (90), possibly through the degradation of IL-33 (91). The effect on eosinophils specifically includes the suppression of apoptosis and induction of chemokine production (92).

Secondary to pre-stored granule proteins, mast cells synthesize eicosanoids within minutes and cytokines, chemokines, and growth factors in a matter of hours. Eicosanoids like the arachidonic acid metabolites prostaglandin (PG) D2, LTB4, LTC4, hydroperoxy-eicosatetraenoic acid, and hydroxy-eicosatetraenoic acid (5-HETE) influence eosinophil trafficking and function in asthma and allergic diseases (93, 94). The early CysLTs and PGD2 prime ILC2s by upregulating cytokine receptors that respond to the epithelial cell-derived cytokines IL-33 and IL-25 (62). An intravital microscopy study in mice showed that IL-33 induced CCR8^+^ ILC2s to patrol the peribronchial and perivascular spaces, possibly localizing eosinophil recruitment to CCL8-rich sites of inflammation (59). Of the prostaglandins, PGD2 can directly bind its receptor DP2/CRTH2 on eosinophils (95, 96), which is robustly expressed on both murine and human eosinophils (97). Exposure of eosinophils to PGD2 induces both activation and chemotaxis (98, 99). Activated mast cells further promote the migration of DCs to the draining LN, contributing to the initiation of adaptive immunity (100). Mast cells further produce cytokines, like IL-3, TNF, IL-4, IL-8, IL-13 and IL-25 (44) and especially, mast cell-derived IL-3 is suggested to play a key role in modulating eosinophil functioning in allergic asthma (101). In fact, IL3 polymorphisms have been associated with decreased risk of asthma (102). In both asthmatic and non-allergic lung eosinophilia, IL-3 production by type 2 CD8^+^ (Tc2) cells is found to be increased (103, 104). However, it is unclear at which stage in the allergic response CD8^+^ Tc2 cells are a significant source of IL-3 for eosinophils.

The highly coordinated acute response to allergens at the epithelial barrier seems to set the stage for the downstream allergic inflammatory eosinophilia. However, the immediate response of lung resident eosinophils or the early infiltration of circulating eosinophils upon antigen challenge are poorly investigated, with most studies investigating time points often days after the last antigenic challenge. The early inflammatory landscape is coordinated by epithelial cells, mast cells, ILC2 and neurons, and their products will primarily target resident eosinophils. At the same time, basophil-derived IL-4 and ILC2-derived IL-5, as well as eotaxin, recruit eosinophils from the periphery. However, upon arrival in the lung the inflammatory input for those cells has changed. It is unknown how the lung resident and infiltrating eosinophils coordinate this response. In addition, the type and combination of inflammatory triggers in the lung, may affect the granulocytic composition. For example, early (30 minute) recruitment of CD101- eosinophils after LPS instillation in the lung, suppress neutrophilic lung inflammation (105). However, combined with HDM, LPS induced neutrophil-derived cytoplasts and neutrophil extracellular trap (NET) formation in the broncho-alveolar lavage (BAL) after 24 hours and increased Th17 cells in the lung-draining lymph nodes (106). The acute phase response is mostly “outward-in”; however, some immediate immune reactions can be found in the alveolar space. Within the alveolar space of allergic asthmatic patients’ segmental challenge with ragweed showed increases in histamine, PGD2 and thromboxane B2 within 5 minutes of exposure (107). Although, no cellular changes were yet observed at this early timepoint. It will be exciting to see how the division of labor between resident and incoming eosinophils is established and how this shapes the propagation of the allergic response.

### Late Phase (Hours to Days)

As local innate inflammation progresses into the late phase, the tissue-contained response is joined by innate cells from the circulation, including neutrophils, eosinophils, basophils and monocytes (44). The lung endothelium is conditioned to allow the tethering, rolling and extravasation of leukocytes into the lung tissue (108). Apart from the chemo-attractants discussed above, endothelial priming is induced by several cell types, like NKT cells and basophils. Whereas mast cells reside in the lung tissue, the majority of basophils are recruited from the periphery into the lung after allergen challenge and affect eosinophil recruitment and function (109, 110). In the late phase of the asthmatic response, basophils are the major IgE bearing granulocyte producing histamine (111). Basophils can, like mast cells, produce IL-3 in response to IgE/FcϵR triggering and facilitate an autocrine activation loop (112, 113). However, as reported above, IL-3 actively affects eosinophil function. In addition to their pro-inflammatory function, murine basophils also prime lung ILC2s to respond to the neuropeptide neuromedin B, possibly to inactivate type 2 immune responses and to aid resolution (114). Moreover, through production of lipoxygenases and cyclooxygenases, mast cells and basophils can balance the metabolism of arachidonic acid into leukotrienes and prostaglandins. For example, allergen/IgE-stimulated bone-marrow-derived basophils were found to secrete 5-lipoxygenase (5-Lox) metabolites LTB4 and LTC4 within 30 minutes of exposure (115). In turn, cyclooxygenase (COX)-metabolites like PGD2 and PGE2 were secreted 6 hours after stimulation. This temporal separation adds to their ability to modulate immune responses and the recruitment of immune cells like eosinophils.

Recruitment of circulating eosinophils into the inflamed lung seems to be regulated at several levels. In asthmatic patients, basophil-derived IL-4 is the cardinal cytokine for recruitment of eosinophils into the lung and was found in the bronchoalveolar lavage within 20 hours after segmental allergen challenge (116). The secreted IL-4 could in turn induce a dose and time-dependent increase in the levels of eotaxin mRNA within fibroblasts (117). Additionally, eotaxin-3 expressed by IL-4-stimulated human vascular endothelial cells may contribute to CCR3-dependent eosinophil accumulation in the lung (118). Similarly, human endothelial cells stimulated with IL-4 increased the expression of VCAM-1, which binds to eosinophil VLA-4 contributing to eosinophil extravasation after allergen challenge (119). Otherwise, Coyle and colleagues reported that IL-4 neutralization just before allergen challenge had little effect on eosinophil infiltration, suggesting that although IL-4 is required for the induction of Th2 immunity, it may be dispensable for eosinophil recruitment in the challenge phase (120). Recently, Felton and colleagues have shown that eosinophil recruitment into tissues is intrinsically dependent on expression of the Ikaros zinc-finger family transcription factor IKZF3 (Aiolos), as Aiolos-deficiency reduced eosinophil CCR3 expression, and subsequent CCL11-induced intracellular ERK1/2 signaling (121). Yi et al. have further shown that it is a network of cDC2s that converge on lung cDC1s, which produce CCL17 and CCL22, directly attracting CCR4-expressing eosinophils (122). Interestingly, the early recruitment within a day was mediated by CD24^-^ cDC2s producing nitric oxide affecting cDC1 activation, whereas eosinophil recruitment was aborted *via* TGFβ-producing CD24^+^ cDC2s in later phases of the inflammatory response (122). CCL17 and -22 not only affect eosinophils, as these chemokines are reported to actively recruit T cells to the lungs sustaining type 2 inflammation (123). The need for eosinophils to induce T cell infiltration in allergic airway inflammation was further corroborated (124), although this prerequisite might be less pronounced in BALB/c mice (125, 126). It is likely that the division of labor by DCs in the lung upon inflammation is tightly coordinated in a spatiotemporal manner to allow DC emigration, T cell activation in the lymph node and T cell recruitment to the lung, but also supply the lung parenchyma with the proper inflammatory context. Circulating eosinophils, resident eosinophils, recruited eosinophils, and bone marrow eosinopoiesis should ideally be analyzed independently, since they are likely functionally different or at least different in their susceptibility to external input. *In vivo* challenge studies in mild asthmatic patients have shown eosinophil-specific changes in the BAL transcriptome 48 hours after segmental bronchoprovocation with allergen (127). These changes may well be induced by infiltrating eosinophils receiving different environmental cues. Possibly, tissue damage is sufficient to induce eosinophil recruitment, as is evidenced by intravital microscopy of lung tissue at 12 hours post silica particle-induced acute injury (17). Moreover, radiolabeled eosinophils injected intravenously into asthmatic patients or healthy volunteers also showed lung infiltration within minutes, with asthmatic patients showed higher eosinophil migration to the lung (128).

Neutrophils also play an important role in allergic lung inflammation and their presence has been related to separate endotypes of asthma. For example, the presence of high bronchial neutrophilia with similar levels of eosinophilia was related to increased serum IgE, IL-17 production and clinical corticosteroid dependence (129). The recruitment of neutrophils in the lung is governed by epithelial club cells under the influence of the circadian rhythm, which mode of attraction underlies the anti-inflammatory capacity of dexamethasone (130). Additionally, neutrophil activation in the form of dsDNA-rich NETosis has been implicated in virally-induced asthma exacerbations of type-2 responses in the acute phase of the immune response (131) (discussed in more detail below). Interestingly, neutrophil depletion in the HDM mouse model exacerbated type 2 inflammation and airway pathology (132). This exacerbation was attributed to increased systemic G-CSF, which activated ILC2 and enhanced antigen presentation by monocyte-derived dendritic cells. In addition, a recent study has shown that IL-17a and TNFs stimulation of lung epithelium resulted in local G-CSF (CSF3) production, leading to increased granulopoiesis and both systemic and respiratory neutrophilia (133). However, the prerequisite of IL-17a and TNF would suggest this process to be relevant in the adaptive phase of the inflammatory response, when helper T cell cytokines are abundant. Increased neutrophilia in asthmatics has been shown to be dependent on epithelial cells and IL-8 derived from smooth muscle cells (134, 135). Although IL-8-mediated neutrophilia may beneficially affect immunity to bacterial lung infection, the resulting extensive lung remodeling may lead to impaired lung function in asthmatics (136).

As the lung tissue recruits cells from the circulation, certain immune cells cross the epithelial barrier to the bronchoalveolar space, including eosinophils, basophils and lymphocytes (107). Within 4 hours after segmental allergen challenge in asthmatic patients, eosinophils are recruited to the alveolar space by epithelial-derived eotaxin (137). Another study in humans found increased ILC2 in the BAL at 24 hours after segmental challenge. Moreover, BAL ILC2 expressed higher levels of IL-13 transcript relative to blood ILC2 (138). At the same time, allergen-specific IgE, C3a, C5a and IL-9 accumulates in the BAL (139–141). Allergen-activated macrophages start producing TNF and IL-6 (142). New alveolar macrophages may be partly replenished by monocyte-derived cells attracted by activated epithelial cells (143) and acquire an alternatively-activated phenotype under the influence of basophil-derived IL-4 (144). Besides, asthmatic patients showed increased epithelial-derived MUC5AC levels, at 24 hours after antigen challenge (145).

During the late phase of the immune response, effector cells are mainly structural cells, resident immune cells and infiltrating immune cells from the circulation. In parallel, DCs are activating allergen-specific T cells in the lymph nodes, which expand and travel to the lung tissue to introduce the adaptive phase.

### Adaptive Phase (Days to Weeks) and Resolution of Inflammation

Following the initial release of type 2 cytokines in the lung (by ILC2 and NKT cells), activated eosinophils upregulate multiple cell surface receptors, allowing them to become dynamically regulated and in turn drive the production of canonical Th2 cytokines IL-4, IL-5, and IL-13 by T cells (146). A systematic investigation of these cytokines revealed single and synergistic effects on eosinophils and lung inflammatory hallmarks, such as goblet cell metaplasia (147). Over time, plasma cells arise. Meanwhile, in the bone marrow eosinophil-derived APRIL and IL-6 have been shown to sustain the survival of co-localizing plasma cells (148). The adaptive phase of the allergic immune response is further characterized by the influx of Th2 cells. Asthmatic patients who clearly present with allergen-specific Th2 cells, and their associated cytokines, in the bronchoalveolar lavage, as well as with airway and/or blood eosinophilia are clustered as type 2-high patients (149, 150). On the other side, non- or low-type 2 asthma phenotypes have also been recognized and are defined by the absence of Th2 cytokine signatures and eosinophilia. However, within the type 2-high subtype, circulating Th2 cells appear to be more diverse than initially expected. Circulating allergen-specific Th2 cells have been identified in the lungs of mice and in the blood of allergic asthmatic patients. These Th2 cells have been found to not only produce IL-4, IL-5, IL-6, IL-13, but also IL-9, IL-17, and IL-21 (151, 152). Th2 cells isolated from both humans and mice are characterized by the expression of PPARγ, that seems to be crucial in driving Th2 cell pathogenicity (152, 153). It seems that IL-4 production in the lung is mostly basophil-derived, while IL-4 present in the draining lymph node was T cell-derived (154). Furthermore, Tibbitt and colleagues have shown that while IL-4 may play a more dominant role in the draining lymph node, IL-5 and IL-13 are more prominent T cell cytokines in the lung tissue. This indicates that Th2 cells undergo substantial programming in the lung, making them highly distinct from their lymph node counterparts (152). We found that IL-21 produced by distinct T cell subsets can promote adaptive Th2 cell responses (151). It should be mentioned that the source of IL-13 in allergen-induced airway hyperresponsiveness may depend on the age of first exposure, with IL-13^+^ CD4^+^ T cells dominating in neonatal life and IL-13^+^ ILC2s dominating in adult mice (155). Interestingly, it has also been suggested that pulmonary NKT cells, which are activated by IL-25, IL-33, and TSLP, can license incoming Th2 cells to induce airway hyperresponsiveness, *via* the production of IL-4 and IL-13 (156, 157). Moreover, Vα14-expressing NKT cells, residing in the intravascular space of the lung microvasculature, can recruit eosinophils after binding of αGalCer on CD1d (158). However, it is not clear how precisely NKT cells drive asthma adaptive immune response.

Whereas the classical type 2 cytokines induce the expression of adhesion molecules, such as ICAM-1 and VCAM1, that allow extravasation of eosinophils into the lung, the function of the more enigmatic cytokine IL-9 is largely unknown (159). Besides being produced by highly-differentiated Th2 cells in allergic asthmatic patients (160), also referred to as Th9 cells (161), IL-9 is also produced by human eosinophils and neutrophils (162, 163). Consequently, increased expression of IL-9 has been found in the bronchoalveolar lavage in these patients (141). Additionally, genome-wide expression profiles showed that young asthmatics with a IL-9 polymorphism were more likely to report a severe asthma exacerbation to HDM (164). In murine models IL-9 seems to be critical for the induction of allergic airway inflammation, as the administration of blocking antibodies reduced asthma features (165). Moreover, TSLP and IL-25 signaling was shown to promote Th9 cell differentiation and stimulated IL-9 production by these cells (166, 167). Elevated levels of IL-9 were further reported to increase mast cell numbers in the lungs. Mast cell precursors are attracted to the lung and seem to peak in numbers one day after a seven-day challenge period (168, 169). In fact, it seems that the Th9 cells are critical to the IL-9-mediated recruitment of late phase mast cells (170). The recruited mature mast cells can persist for weeks post allergen challenge, further reinforcing the Th2 environment. In all, incoming T cells produce high-levels of Th2 cytokines and thereby maintaining and propagating asthma features, including the recruitment of eosinophils into the lungs and airways. Samples derived from human asthmatics showed that eosinophils may further sustain Th2 inflammation by maintaining high indoleamine 2,3-dioxygenase (IDO) levels (171). Interestingly, pulmonary eosinophil trafficking into the lung lymphatic compartment is shown to be dependent on LTC4 (172), but independent of eotaxin (34), and has been proposed to be a prerequisite for DC accumulation in the draining lymph nodes and allergen-specific T cell proliferation (39). As mentioned during the sensitization phase, eosinophils can migrate to the draining lymph nodes and localize to the T cell-rich paracortical areas. During the adaptive phase of the allergen-induced immune response, eosinophils have been shown to stimulate antigen-specific T cell proliferation within the lymph nodes (34). Additionally, eosinophils have the ability to influence proliferation and activation of both memory T and B cells, yet have little effect on naïve T and B cells. Interestingly, eosinophilic airway inflammation was unaffected in a chronic HDM model in the absence of B cells or CD40L-dependent B-T cell interactions (173). As the blood-derived eosinophils infiltrate the inflamed lung, the circulation is replenished through increased granulopoiesis in the bone marrow. While the relationship between systemic infection and emergency neutrophil output from the bone marrow is well established (174), it is unclear how acute or chronic allergic lung inflammation affect eosinopoiesis in the bone marrow. IL-5 is clearly the most important factor promoting eosinophil production, differentiation, and in preventing apoptosis (1). Despite the fact that the developmental pathway of eosinophils has been reviewed extensively, its precise trajectory under inflammatory conditions is still a matter of debate (175). Nonetheless, it is worth noting that allergic lung inflammation leads to increased eosinopoiesis *via* systemic IL-5 and further differentiation *via* systemic or local IL-3, GM-CSF and eotaxins (CCL11, CCL24, CCL26) (176).

Although often considered pro-inflammatory, eosinophils have also been suggested to mediate the resolution of inflammation. For example, immune resolution of the airways after allergen exposure is defective in PHIL mice, which lack eosinophils (177). The resolution phase is characterized by apoptosis of various immune cells, and the subsequent uptake by macrophages. Eosinophils can induce macrophage CXCL13 expression in the resolution phase, leading to increased macrophage-dependent phagocytosis and impaired lymphatic drainage (177). Additionally, CXCL13 recruits B cells and CD4^+^ T cells to the lung, where these may contribute to induced bronchial-associated lymphoid tissue (iBALT). In turn, iBALT structures may facilitate or reduce the accumulation of eosinophils in allergic lung inflammation, depending on the timing and the research model used (178). As the innate inflammatory response needs resolution, several inter-/intracellular negative feedback loops exist to resolve inflammation, remodel damaged tissue and instigate tissue repair (179). Granulocytes are thought to travel into the airways, undergo apoptosis and are removed by macrophage (or epithelial) efferocytosis (180). In the lung, IL-4 and IL-13 together with apoptotic cells programs macrophages to go into a tissue repair phenotype (181, 182). This removal of apoptotic neutrophils is mediated by expression of Gas6 and recognition by its cognate receptors AXL and MERTK (183). A similar MerTK-dependent mechanism of efferocytosis of eosinophils has been described in an ovalbumin-induced allergic inflammation model (184). Moreover, a failure to undergo apoptosis through experimental overexpression of the anti-apoptotic protein Mcl-1 resulted in exacerbated allergic airway inflammation (185). Furthermore, in a murine asthma model phagocytosis of apoptotic cells by alveolar macrophages, resulted in the production of retinoic acid, which promoted regulatory T cell development (57). The notion that apoptosis is the major driver of eosinophil removal from the airways is however contested (186). Eosinophils can undergo a specific type of lytic cell death, which involves the expulsion of DNA-contained eosinophil extracellular traps (EET) and granules (187). Alternatively, eosinophils may undergo ferroptosis-like cell death, which may reduce allergic airway inflammation in mice when therapeutically promoted (188). Nonetheless, how eosinophil death is regulated as part of the resolution phase or during chronic inflammation is unclear. If the clearance of dying cells is impaired, apoptotic cells become necrotic and damage-associated molecular patterns are released, which may actually result in additional inflammation. To aid the clearance of death cells, non-professional phagocytes, such as bronchial epithelial cells, can contribute to apoptotic cell clearance and the restoration of homeostasis (189). Both innate and adaptive immune cells are communicating to ensure resolution of inflammation. For example, IL-33 may stimulate mast cells to produce IL-2, which promotes the expansion of Tregs. These Tregs, in turn, suppress the development of papain- or IL-33-induced eosinophilia in the lung (190). However, the exact time and cellular context of IL-2 production will affect the final outcome (191). IL-33 and IL-13 have also been shown to coordinate macrophage-mediated bronchial epithelial cell repair after lung injury (182, 192), as well as the production of amphiregulin by ILC2 (193). The identification of functional heterogeneity in these immune responses, under the influence of the changing local tissue microenvironment, may reflect their differential roles in regulating proinflammatory versus tissue-protective responses. However, in the case of chronic inflammation, the line between adequate immune activation, immune resolution and tissue regeneration remains even less well defined.

### Chronic Phase (Weeks-Years)

Clinical data and investigational reports on mild and severe asthmatic patients provide invaluable information about the chronic phase of the allergic lung. A benchmark study by the groups of Teichmann and Nawijn explored this cellular landscape of the lower airways of healthy and asthmatic lungs by single cell RNA sequencing (194). They revealed a shift in airway structural cell communication to a Th2-dominated interactome in asthmatic lungs compared to healthy lungs. Furthermore, bronchoscopy biopsies from asthmatic patients showed enriched mast cells with high expression of genes involved in downstream biosynthesis of PGD2. Repeated activation of these pulmonary mast cells by allergens in asthma patients can result in lowering their degranulation threshold (195, 196). However, whereas increased mast cell numbers were observed in the bundles of airway smooth muscle from chronic asthmatics, these bundles had a profound absence of T cells or eosinophils (197). Nonetheless, intraepithelial mast cell accumulation is associated with a type 2-high phenotype (198). These mast cells seem to be actively degranulating and may be related to fatal cases of asthma (199). Additionally, in children with severe asthma, mast cells were positively correlated with high numbers of submucosal eosinophils (200). Likewise, a negative correlation was observed between eosinophil counts in atopic individuals and their epithelial barrier integrity (196). Collectively, it seems that the cellular landscape in the lungs of chronic asthmatics is thoroughly affected, resulting in airway dysfunction, as well as mucus- and goblet cell metaplasia.

The relationship between eosinophils and airway dysfunction has been extensively researched. The exact role of eosinophils in common mouse models of allergic airway inflammation likely depends on several factors, including the chronicity of the model, the genetic background, the number of antigenic exposures, the type of allergen, and the mode of antigen delivery (201). Early studies using the OVA protocol with IL-5 knockout mice (202), congenitally eosinophil-deficient mice (203) and eosinophil depleting biologics (204) implicated an important role of eosinophils in airway inflammation. In contrast, Takeda and colleagues showed that an extensive OVA model (2 sensitizations and 7 or 11 challenges over a 50 to 66 day period, respectively) developed airway hyperreactivity reaction (AHR) in both WT and PHIL (eosinophil-deficient) transgenic animals (179). Importantly, eosinophil-deficient animals showed eosinophilic-independent AHR, likely through increased goblet cell numbers after 11 OVA challenges. Jacobsen and colleagues described a genetic mouse model of chronic Th2–driven inflammation by overexpressing IL-5 from T cells and human eotaxin 2 in the lung (*I5/hE2*), which did not show extensive pulmonary histopathology regardless of clear eosinophil activation, type-2 immunity and degranulation (205). Similarly, clinical studies were unable to find an association between reduced eosinophilia by mepolizumab (IL-5 blockade) and airway function/hyperreactivity, although fewer exacerbations were observed (206).

In chronic asthmatics, both prostaglandins and leukotrienes are deregulated and the production of 5-HETE, LTB4 and LTE4 was found to be increased in alveolar macrophages, leading to defective apoptotic cell phagocytosis (207, 208). This may lead to aberrant cell accumulation and increased necroptosis or eosinophil cytolysis. Human eosinophils have also been linked to the deposition of Charcot-Leyden crystals (CLCs), formed after eosinophils undergo cytolysis and form extracellular traps (EET) (209, 210). In severe asthmatics peripheral EET-forming eosinophils are elevated and can stimulate IL-33 and TSLP production by lung epithelial cells (211). Moreover, CLC formation leads to the production of IL-1β, IL-6, and TNF, as well as the recruitment of several innate and adaptive immune cells and the induction of mucus production by epithelial cells (209, 212). CLC crystals are found more abundantly in asthmatic patients, where they are located within the mucus plugs potentially changing their rheology and rigidity, making it harder to cough them up (209, 213). CLC are amply found in patients with chronic rhinosinusitis with nasal polyps (CRSwNP), a condition of type 2 inflammation of the nose and paranasal sinuses. Here, crystals promote neutrophil recruitment and neutrophil NETosis, creating a favorable niche for persistent type 2 immune cells (214). In a recent study in mice EETs were shown to activate PNECs to produce CGRP and GABA, contributing to asthma pathology (215). Finally, EETosis also involves the release of intact granules that retain granule proteins and can still be activated by CCL11 (187). It has been proposed that these bioactive cell-free granules remain pathogenic in the tissue after IL-5/IL-5R blockade, possibly explaining sustained pathology regardless of significant reductions in eosinophil counts.

In both murine experimental asthma models, as well as in patients with eosinophilic asthma, a population of CD4^+^ resident memory T (Trm) cells was observed (194, 216). Trm cells express the IL-33 receptor ST2, suggesting they could be directly activated by epithelial-derived IL-33 and contribute to the chronicity of the asthma pathogenesis (217). Indeed, higher levels of ST2 were found on allergen-specific CD4^+^ T cells in the BAL of asthmatics after segmental allergen challenge (145). In murine models, allergen-specific Trm cells produced more Th2 cytokines than circulating Th2 cells. Interestingly, the functional difference between the pool of lung Trm and circulating memory cells could be further explained by their localization. Whereas circulating Th2 cells preferentially localized in the lung parenchyma, controlling eosinophil and T cell recruitment, Trm cells localized primarily near the airways and induced eosinophil activation, mucus production, and AHR (218). In human tissue samples from CRSwNP, there was a notable expansion of basal cells at the expense of epithelial cell diversity. This process was not only driven by type-2 cytokines (IL-4 and IL-13), but also induced a possible memory-like phenotype in the basal cell population (219). When comparing allergic asthmatics with allergic nonasthmatic controls, both groups developed allergic airway inflammation in response to allergen. However, in the asthmatic patients type-2 cytokine levels and mucin levels were substantially higher compared to controls (145). Interestingly, type-2 cytokine levels only correlated with mucin production in the asthmatic subjects, but not in the controls, suggesting differences in the airway epithelial responses to inflammation (145). Besides, chronic exposure of the lung to IL-33 seems to drive the allergic immune response beyond the typical type 2 phenotype towards aberrant remodeling of lung epithelium and lung parenchyma (220).

Importantly, not only adaptive antigen-specific immune cells like T and B cells are educated by previous inflammatory insults. This suggests that alveolar macrophages, ILC2, and lung epithelial (stem) cells may be functionally and epigenetically reprogrammed by an inflammatory insult or inflammatory microenvironment. However, it remains to be determined whether it is the same alveolar macrophage lineage or newly recruited monocyte-derived alveolar macrophages, that are subject to this environmental imprinting. In settings of allergic airway inflammation, papain and IL-33 have been shown to induce long-term changes in lung ILC2, with some persisting up to two months in the lung and even 4 months in the mLN. Exposure of these ‘conditioned’ ILC2 to a second unrelated allergen resulted in exaggerated cytokine responses and increased type 2 immune response (221). Interestingly, ILC memory resembles adaptive T cell memory even in absence of antigen-specificity. A novel finding is the presence of inflammatory memory in basal cells from allergic nasal polyp samples (222). Basal cells were found to expand at the expense of differentiated epithelial cells and displayed IL-4/IL-13 responsive genes that remained fixed *ex vivo*. It is unknown whether eosinophils or granulocyte progenitors display such immunological memory as a result of chronic allergic airway inflammation. However, it is clear that chronic asthma patients are immunologically predisposed to airway insults that result in repeated acute and adaptive immune responses aimed at antigen clearing and tissue repair.

Like all chronically ill individuals, asthmatic patients inevitably enter the clinic with an extensive inflammatory history involving chronic eosinophilia. Experimental eradication of eosinophils in animal models before onset of chronic inflammation severely compound the conclusions that can be extrapolated in relation to eosinophil functioning in asthmatics. Another observation of interest is the shortening of telomere length in peripheral leukocytes of asthmatics (223, 224), suggesting extensive leukocyte proliferation and found to correlate with eotaxin 1 expression (225). However, telomerase-deficient mice showed debilitating eosinophil responses in the lung and reduced eosinopoiesis, although eosinophil-independent effects of telomerase cannot be excluded (226). Whether constant eosinopoiesis in long-term severe asthmatics induces inflammaging phenotypes in eosinophils remains unknown.

## Eosinophils in Asthma Exacerbations

Chronic asthmatics are commonly hospitalized for asthma exacerbations and these account for roughly one-third of all asthma-related deaths in the US (227). Exacerbations of asthma can be induced by various different stimuli, including allergens, pollution, cold air, microbes, and viruses. Amongst the latter, respiratory viruses and especially respiratory syncytial virus (RSV) and rhinovirus (RV) are major drivers of asthma exacerbations in children and adults, respectively (228). Respiratory viruses most frequently infect lung epithelial cells (229). Remarkably, asthma exacerbations are mainly induced in patients with high eosinophil numbers (type 2 high phenotype) (230). Recent studies have shown that during viral-induced asthma exacerbations, high levels of IL-33 were produced by airway epithelial cells, consequently suppressing type-I IFN production and leaving the epithelium more vulnerable to repeated infections (231–233). Likewise, epithelial cells from asthmatics showed defective interferon λ production after infection with rhinovirus (234). Importantly, a recent study suggests that the response of the lung epithelium to rhinovirus infection is not qualitatively changed, but is delayed (235). The epithelium of healthy patients showed a peak in the anti-viral response at 48 hours post-infection, whereas lung epithelial cells from asthma of COPD patients peaked at 96 hours post-infection (235). As exposure to respiratory viruses increased the levels of IL-33 and TSLP produced by lung ECs, it comes to no surprise that children hospitalized with severe respiratory infection had increased ILC2 numbers in the lungs (236). Equivalently, in murine models of influenza infections, increased numbers of ILC2 were found in the lungs. Although influenza is rarely involved in asthma exacerbations, these data may suggest a division of labor between Th2 cells contributing early in the response and ILC2-derived cytokines that contribute at a later stage to lung repair *via* the production of amphiregulin (237). Recent studies have also identified a specific population of SIRPα^+^IFNAR^+^ conventional DC2 with strong capacities to activate antiviral CD4^+^ and CD8^+^ T cell responses (238). Such DC responses are dependent on type-I interferons, which are high during antiviral responses and are known to inhibit ILC2 functions (239). However, within the Th2 environment, the levels of type-I interferons may be lower and this may impact the function of SIRPα^+^IFNAR^+^ DC2s and the subsequent antiviral response. It is still unclear how exactly type-I interferons, SIRPα^+^IFNAR^+^ DC2s, and virus-induced asthma exacerbations are linked. Nonetheless, it is tempting to speculate that increased levels of IL-33, produced by airway epithelial cells upon respiratory viral infection, and stronger activation of Th2 cells and ILC2, would enhance asthmic features, including BHR and eosinophilia.

A common feature of the asthmatic lung is the disruption of the airway epithelium (240). An increase of epithelial cells in the sputum (sometimes referred to as Creola bodies) of pediatric asthma patients was found during acute exacerbations (241, 242). These exacerbations were related to increased IL-8, which recruits neutrophils and in turn eosinophils to the lungs (243). Similar findings were reported in a model of rhinoviral-induced asthma exacerbation (244), with type 2 cytokines potentially enhancing the epithelial production of CXCL10, IL-8 and GM-CSF (245). Of note is the observation that the immune response to RV is changed by mepolizumab, without directly affecting eosinophil functioning (246). Taken together, it is clear that both neutrophils and eosinophils enhance RV exacerbations in asthmatics (243).

Human eosinophils express several functional Toll-like receptors (TLRs), including TLR1, 2, 3, 4, 5, 6, 7, and 9, with some heterogeneity associated with atopic status (247, 248). Besides, supporting a potential role for eosinophils in PAMP recognition, TLR expression can provide a mechanism by which bacterial or viral infections exacerbate allergic disease (249). However, to complicate things, eosinophils may play an important role in the protection against viral and bacterial pathogens. Mouse studies with the murine equivalent of RSV, pneumonia virus of mice indicated that eosinophil degranulation was associated with a more favourable outcome in infected mice (250). Although, in human rhinovirus infection, eosinophils were found to lower epithelial interferon production, thereby increasing viral load (251). In patients with RSV infection, eosinophil degranulation products, such as ECP and EDN, have been isolated from the bronchoalveolar lavage of the lower airways (252). More recently, it has been shown that EDN can enter viral capsids and degrade RNA from RSV (253). Eosinophils have even been shown to quickly internalize and inactivate RSV and influenza virus *in vitro*; a characteristic that was defective in eosinophils from asthmatics (254). From murine studies, it is clear that mice overexpressing both IL-5 and eotaxin-2 were protected against lethal pneumovirus infection (250). Eosinophil-driven antiviral activity has further been demonstrated for other respiratory viruses, including influenza, parainfluenza, and HIV, although the exact mechanisms by which eosinophils protect from viral infections have still to be elucidated (253, 255, 256). Taken together, there is a complexity within eosinophil function in viral infection and it is unclear how eosinophil-viral interactions are regulated. As the majority of the eosinophil-viral interactions comes from RSV research, the investigation of other viruses, like rotavirus or SARS-CoV2, may provide further insights regarding eosinophil-viral interactions.

## Concluding Remarks and Future Directions

Our perspective on the lung has changed dramatically over the last decades, culminating in the view of the lung as a place where epithelial cells, stromal cells, and immune cells support a multifaceted frontline defense system focused on inducing tolerance, supporting highly-efficient injury-repair responses, as well as (destructive) inflammatory responses, like asthma. Indeed, not a single cell seems to be left out of the inflammatory response to airborne allergens. Over the last years, mouse models of asthma have evolved from primarily focusing on the role of eosinophils as proinflammatory cells, to a consensus that eosinophils have a divers set of functions ranging from proinflammatory to immune modulating. In these nuanced disease settings, it can be questioned whether, where, and when eosinophils are contributing cells, rather than primary mediators. Shifting the focus of eosinophils being the primary promotors of the inflammatory cascade, towards a view where eosinophils play multiple defined roles along the disease progression trajectory. This may explain the mixed results of eosinophil-depleting therapies in asthma and other inflammatory diseases. The traditional view of eosinophils as being released into circulation as terminally differentiated cells led us to ask the question at which level within the developmental pathway functional differentiation and plasticity is arranged. Recent findings on other granulocytes like neutrophils have addressed similar questions on plasticity in the overarching developmental trajectory and into inflammatory situations. For example, certain combinations of transcription factors govern specific parts of the neutrophil inflammatory response (257). Interestingly, neutrophils are released into circulation in a circadian rhythm and a recent study showed that this chronicity (termed “neutrotime”) largely determines the core transcriptional profile of neutrophils (258), with limited transcriptional change inducible by external input. Whereas these significant advances are made possible using scRNA sequencing, the eosinophil is notoriously difficult to capture in single cell transcriptomics. Hypotheses that are currently entertained include the possibility that RNAses are abundant present in eosinophils and may break down mRNA before it can be amplified, and the terminally differentiated status that simple excludes active transcriptional plasticity. A recent study using the 10X scRNAseq platform showed that the transcriptome of circulating eosinophils is very low, even though eosinophils can be readily detected by RNA-barcoded antibodies in the same setup (CITEseq) (259). Even if transcriptional changes can be found in eosinophils between conditions of *in vivo* allergen challenge in asthmatics, it is unclear whether the readout arises from transcriptional changes in resident eosinophils or the transcriptionally pre-activated circulating eosinophils infiltrating the inflamed lung. The study from Mesnil and colleagues support the latter option, where “inflammatory” eosinophils are proposed to accumulate independent of resident eosinophils. This raises the question at which level local adaptation can occur; are eosinophils victims of predetermined signaling cascades or can they still change their core programs upon receiving environmental cues?

The modulation of eosinophils in the allergic lung, and beyond, may otherwise entail post-transcriptional changes like metabolic switches or translational modifications. We have recently shown that eosinophils participate in the competition for glucose in the tumor microenvironment of lung metastases, inhibiting anti-tumor NK cells (260). Interestingly, eosinophils appear to display greater metabolic flexibility compared to neutrophils, and can switch metabolic programming during *in vitro* differentiation. Hence, eosinophil swarming in the allergic lung will undoubtedly affect local immunometabolism (261, 262).

Current eosinophil depleting strategies may pose, currently unknown, pre-dispositions to other diseases. Thus warranting a more sophisticated approach to modulating their function. If we are to understand eosinophil functioning in space and time, we will undoubtedly need to resort to new and more refined modes of measuring eosinophil states along the developmental trajectory. As the last frontier in myeloid developmental understanding on the single cell level, the eosinophil may yet prove to be a new dimension.

## Author Contributions

SS and MS conceptualized and wrote the manuscript. All authors contributed to the article and approved the submitted version.

## Funding

We acknowledge the following funding sources for SS; Dutch Research Council Rubicon (452019321), Fonds Wetenschappelijk Onderzoek Vlaanderen/F.R.S.-F.N.R.S. EOS consortium U-HEAD, and for MS; Fonds Wetenschappelijk Onderzoek Vlaanderen (12Y5322N), Fund Suzanne Duchesne (managed by the King Baudouin Foundation), and Fondation ACTERIA.

## Conflict of Interest

The authors declare that the research was conducted in the absence of any commercial or financial relationships that could be construed as a potential conflict of interest.

## Publisher’s Note

All claims expressed in this article are solely those of the authors and do not necessarily represent those of their affiliated organizations, or those of the publisher, the editors and the reviewers. Any product that may be evaluated in this article, or claim that may be made by its manufacturer, is not guaranteed or endorsed by the publisher.
